# Rabbit haemorrhagic disease virus 2 from Singapore 2020 outbreak revealed an Australian recombinant variant

**DOI:** 10.1093/ve/vead029

**Published:** 2023-05-02

**Authors:** Eileen Y Koh, Jasmine Ong, Yifan Wang, Xinyu Toh, Charlene Judith Fernandez, Taoqi Huangfu, Robyn N Hall, Steffie Toh, Kelvin Lim, Wendy Sng, Hwee Ping Lim, Kelvin Ho, Siow Foong Chang, Him Hoo Yap

**Affiliations:** Centre for Animal & Veterinary Sciences, Professional and Scientific Services, Animal and Veterinary Service, National Parks Board (NParks), 1 Cluny Road, Singapore Botanic Gardens 259569, Singapore; Centre for Animal & Veterinary Sciences, Professional and Scientific Services, Animal and Veterinary Service, National Parks Board (NParks), 1 Cluny Road, Singapore Botanic Gardens 259569, Singapore; Centre for Animal & Veterinary Sciences, Professional and Scientific Services, Animal and Veterinary Service, National Parks Board (NParks), 1 Cluny Road, Singapore Botanic Gardens 259569, Singapore; Centre for Animal & Veterinary Sciences, Professional and Scientific Services, Animal and Veterinary Service, National Parks Board (NParks), 1 Cluny Road, Singapore Botanic Gardens 259569, Singapore; Centre for Animal & Veterinary Sciences, Professional and Scientific Services, Animal and Veterinary Service, National Parks Board (NParks), 1 Cluny Road, Singapore Botanic Gardens 259569, Singapore; Centre for Animal & Veterinary Sciences, Professional and Scientific Services, Animal and Veterinary Service, National Parks Board (NParks), 1 Cluny Road, Singapore Botanic Gardens 259569, Singapore; Health and Biosecurity, Commonwealth Scientific and Industrial Research Organisation, Clunies Ross Street, Acton, Australian Capital Territory 2601, Australia; Centre for Invasive Species Solutions, University of Canberra, Bruce, Australian Capital Territory 2617, Australia; Centre for Animal & Veterinary Sciences, Professional and Scientific Services, Animal and Veterinary Service, National Parks Board (NParks), 1 Cluny Road, Singapore Botanic Gardens 259569, Singapore; Veterinary Health Management, Professional and Scientific Services, Animal and Veterinary Service, National Parks Board (NParks), 1 Cluny Road, Singapore Botanic Gardens 259569, Singapore; Veterinary Health Management, Professional and Scientific Services, Animal and Veterinary Service, National Parks Board (NParks), 1 Cluny Road, Singapore Botanic Gardens 259569, Singapore; Veterinary Health Management, Professional and Scientific Services, Animal and Veterinary Service, National Parks Board (NParks), 1 Cluny Road, Singapore Botanic Gardens 259569, Singapore; Veterinary Health Management, Professional and Scientific Services, Animal and Veterinary Service, National Parks Board (NParks), 1 Cluny Road, Singapore Botanic Gardens 259569, Singapore; Professional and Scientific Services, Animal and Veterinary Service, National Parks Board (NParks), 1 Cluny Road, Singapore Botanic Gardens 259569, Singapore; Animal and Veterinary Service, National Parks Board (NParks), 1 Cluny Road, Singapore Botanic Gardens 259569, Singapore

**Keywords:** RHDV, Singapore, lagovirus, calicivirus, genomic epidemiology

## Abstract

Rabbit haemorrhagic disease (RHD) is a significant and debilitating viral disease affecting lagomorphs. In September 2020, Singapore reported its first cases of RHD virus (RHDV) infection in domesticated rabbits. The initial findings reported that the outbreak strain belonged to genotype GI.2 (RHDV2/RHDVb), and epidemiological investigations could not identify the definitive source of the virus origin. Further recombination detection and phylogenetic analyses of the Singapore outbreak strain revealed that the RHDV was a GI.2 structural (S)/GI.4 non-structural (NS) recombinant variant. Sequence analyses on the National Centre for Biotechnology Information (NCBI) database showed high homology to recently emerged Australian variants, which were prevalent in local Australian lagomorph populations since 2017. Time-structured and phylogeographic analyses for the S and NS genes revealed a close genetic relationship between the Singapore RHDV strain and the Australian RHDV variants. More thorough epidemiological inquiries are necessary to ascertain how an Australian RHDV was introduced into the Singapore rabbit population, and opportune development of RHDV diagnostics and vaccines will be important to safeguard lagomorphs from future RHDV infection and disease management.

## Introduction

For more than four decades, global populations of both wild and domesticated European rabbits were ravaged by the highly fatal and contagious rabbit haemorrhagic disease (RHD) (Liu et al. 1984; Abrantes et al. 2012), causing substantial economic losses in the rabbit meat and fur industry and significant impacts to wild populations and their dependent predators, whether direct or indirect (Delibes-Mateos et al. 2007). Symptomatic manifestations of RHD include respiratory signs, fever, loss of appetite, and lethargy (Marcato et al. 1991); these can be presented as three clinical forms—per-acute, sub-acute, and chronic—which could lead to either high case fatality or recovery with acquired immunity (Xu and Chen 1989). A peracute course with a fatal outcome is observed in >95 per cent of cases in naive animals.

RHD is caused by a non-enveloped icosahedral virus with a positive-sense single-stranded ∼7.4-kb RNA genome belonging to the *Lagovirus* genus of the *Caliciviridae* family. *Lagoviruses* are genotypically classified by their major capsid protein (VP60) and, to a secondary extent, by their polymerase types (Le Pendu et al. 2017) into genogroups (e.g. GI and GII), genotypes (e.g. GI.1, GI.2, and GI.4), and variants (e.g. GI.1a, GI.1b, and GI.1c) (Le Pendu et al. 2017). The first reported lagovirus, now classified as European Brown Hare Syndrome Virus (EBHSV (genogroup GII), was reported in European brown hares (*Lepus europaeus*) in the early 1980s (Gavier-Widén and Mörner 1993); a related virus, RHD virus (RHDV (genotype GI.1)), emerged in European rabbits (*Oryctolagus cuniculus*) around the same time, which subsequently spread to China and the rest of Europe (Xu and Chen 1989; Abrantes et al. 2012). During the mid-1990s, antigenic variants of RHDV (RHDVa or genotype GI.1a) were reported. Subsequently, in 2011, RHDV2 (genotype GI.2) was first reported in France (Le Gall-Recule et al. 2011) and spread rapidly throughout the global lagomorph population. Although lagomorphs are the only biological host for RHDV, the virus can be spread mechanically by flies (Asgari et al. 1998), predators, fomites, and sympatric mammals (Bao et al. 2020; Abade Dos Santos et al. 2022).

In recent years, recombination events have been found to be extremely common in lagoviruses, with a major recombination breakpoint at the junction of non-structural (NS) and structural (S) genes (Mahar et al. 2021). The structural proteins (VP60 and VP10) are the primary determinants of host range, antigenicity, and pathogenicity (Mahar et al. 2021). The pathogenic and non-pathogenic forms of RHDV were observed over the years, with reports suggesting that the pathogenic lagoviruses emerged either through direct evolution from a benign ancestor or through a species jump (Kerr, Kitchen, and Holmes 2009; Esteves et al. 2015). An example of these benign lagoviruses is GI.4. This is a benign enterotropic virus, also known as rabbit calicivirus-A1, which has been reported in the 1950s in both wild and domesticated rabbit populations (Mahar et al. 2016). However, its geographical distribution in Australia is not as significant as that of RHDV (Liu et al. 2014). Other benign enterotropic lagoviruses have also been reported from elsewhere (Capucci et al. 1996; Nicholson et al. 2017; Lemaitre et al. 2018).

In 2020, an outbreak of RHDV was reported in Singapore for the first time, where thirteen domestic pet rabbits from three different households were observed to exhibit varying degrees of appetite loss and elevated liver enzymes prior to their demise (Toh et al. 2021). Positive reverse-transcription polymerase chain reaction (PCR) amplification of the RHDV VP60 gene was obtained (Le Gall-Reculé et al. 2013). Molecular characterisation of the viral genomes using the sequence-independent, single-primer approach on the Illumina iSeq100 platform revealed the infecting variant to be of genotype GI.2 (RHDV2/RHDVb) (Toh et al. 2021). Subsequent epidemiological investigations ruled out viral introduction via the importation of infected rabbits and contaminated feed. It could be shown that the virus had spread both within and across households and veterinary clinics despite the strict local biosecurity, border control measures, and absence of wild rabbit populations in Singapore (Lim et al. 2021). The route of incursion has not been determined at the time of writing so far.

To determine if the Singapore RHDV variant underwent recombination and/or mutation, the authors further characterised the virus isolated from the liver of a rabbit that died in Singapore in 2020 (as reported in an earlier study by Toh et al. 2021) using time-structured phylogenetic and phylogeographic analyses.

## Materials and methods

### Samples and genetic sequencing

The sequence (also known as NParks/M54-9 herein, GenBank nucleotide accession number MW194928) used in this study was generated from an RHDV-infected rabbit, as previously reported by Toh et al. (2021). Briefly, the sequence was derived from the extracted RNA and from next-generation sequencing on the Illumina iSeq100 platform (Toh et al. 2021). No ethical approval was required.

### Recombination analysis

To determine whether recombination events occurred, the complete coding sequence was aligned with that used by Abrantes et al. (2020), representing genotypes GI.1, GI.2, GI.3, and GI.4. The final data set comprising 225 sequences and 7,370 nucleotides in length was screened for recombination with the Recombination Detection Program version 5 (RDP5) using the recommended methods within the program and coupled with analysis parameters as described by Abrantes et al. (2020). If at least three out of seven detection methods showed a statistically significant difference (*P* value of <0.05), the sequence was considered a potential recombinant sequence (Martin et al. 2021).

### Phylogenetic analysis

Near-complete lagovirus sequences spanning the known genetic diversity of this genus were retrieved from the NCBI nucleotide database and downsampled using CD-HIT-EST (Li and Godzik 2006) based on a 95 per cent nucleotide identity cut-off. The 2020 Singapore RHDV sequence (GenBank accession number MW194928) was aligned with these representative lagovirus sequences using the FFT-NS-2 algorithm as implemented in Multiple Alignment using Fast Fourier Transform (MAFFT) v7.450 (Katoh and Standley 2013). The alignment (*n* = 127 sequences) was trimmed and subdivided into NS and S regions (nucleotides 4–5265 and 5266–7347 of MW194928, respectively); maximum-likelihood phylogenies were estimated separately for each of these regions using IQTREE2 v2.1.2 (Minh et al. 2020), with the best-fit model as selected by ModelFinder (Kalyaanamoorthy et al. 2017). Branch support was estimated with 1,000 ultrafast bootstrap replicates (Hoang et al. 2018) and 1,000 replicates of the SH-aLRT test (Guindon et al. 2010). Phylogenies were rooted at the midpoint between the genogroup I and II clades.

### Time-structured phylogeographic analysis

We retrieved all lagovirus sequences over 1,000 nucleotides in length from GenBank and aligned these using MAFFT v7.490 (Katoh and Standley 2013), as implemented in Geneious Prime 2022.2.1. For the VP60 S data set, we extracted all GI.2 sequences from this alignment; for the NS data set, we extracted all GI.4 sequences. We excluded sequences that did not have a collection date and location available. For the VP60 data set, we restricted the number of Australian sequences to reduce bias due to the relatively high sampling rate of lagoviruses in Australia. The extracted alignments were manually trimmed. We included NC_002615 (the reference sequence of the EBHSV) in both alignments as an outgroup. The final GI.2 VP60 alignment was 1,743 nucleotide (nt) in length and comprised 293 sequences; the final GI.4 NS alignment was 5,268 nt in length and comprised 140 sequences. The taxa included within each data set are detailed in Supplementary Table S1.

For each alignment, a maximum-likelihood phylogeny was inferred using IQTREE v2.0.3 (Minh et al. 2020), with automatic model selection using ModelFinder (Kalyaanamoorthy et al. 2017); branch support was assessed through 1,000 ultrafast bootstrap approximations (Hoang et al. 2018) and 1,000 iterations of the SH-aLRT test (Guindon et al. 2010). This phylogeny was used as input to generate a time-structured phylogeny using TreeTime (Sagulenko, Puller, and Neher 2018). TreeTime has been shown to achieve similar or better accuracy than other phylodynamic methods when estimating clock rates for low-diversity data sets, as is the case with these data (Sagulenko, Puller, and Neher 2018). We used a constant coalescent rate and uncorrelated clock with a model inferred from the data and accounting for covariation. These priors have previously been determined to be suitable for RHDV (Mahar et al. 2021). The clock filter was set to off. The GI.2 VP60 data set was run for five iterations (and converged after two), while the GI.4 NS data set was run for 1,000 iterations and, however, still did not reach convergence.

For ancestral state reconstruction of the geographical location (i.e. phylogeography) of branches and internal nodes, we used the TreeTime migration function with the time-structured phylogeny as input (Sagulenko, Puller, and Neher 2018).

Trees were plotted in R v4.1.3 (R Core Team 2021) using the tidyverse v1.3.2 (Wickham et al. 2019), ggtree v3.2.1 (Yu et al. 2017), and plotly v4.10.1 (Sievert 2020) packages and polished in Inkscape v1.1.2.

## Results

### Recombinant analysis of RHDV gene fragments

**Figure 1.**
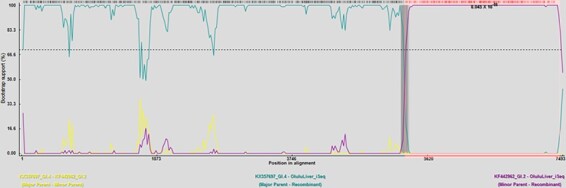
BootScan plots of RHDV Singapore variant (as query), compared with published RHDV strains, showing potential recombination events. Sudden alterations in bootstrap support indicate recombination. A single recombination breakpoint was determined at the NS/S boundary located at the nucleotide 5,314 position according to MW194928. The parental strains were identified as GenBank accession numbers KX357697 (GI.4; top) and KF442962 (GI.2; bottom) . The reference sequences were retrieved from Abrantes et al. (2020).

The sequence alignment was screened for recombination using the RDP software (*n* = 225 and 7,370 nucleotides). All seven methods (i.e. RDP, GENECONV, BootScan, MaxChi, Chimaera, SiScan, and 3Seq) available in the RDP software detected the 2020 Singapore RHDV strain as a recombinant with strong statistical support (*P* < 0.05) (Table 1). A single recombinant breakpoint was determined at nucleotide position 5239–5357 basepairs, corresponding to the NS/S junction (Fig. 1). The RHDV AUS/VIC/BEN-115/2010 (GenBank accession number KX357697) (Mahar et al. 2016) and Algarve3_Portugal (GenBank accession number KF442962) (Lopes et al. 2015) were identified as NS and S donors, respectively, based on the sequences included in the analysis (Fig. 1).

**Table 1.** Results of the RDP analysis.

		Likely donor strain		Methods and average *P* values						
Strain	Recombination breakpoint (nucleotide position) at 99% CI	Non-structural genes	Structural genes	RDP	GENECONV	BootScan	MaxChi	Chimaera	SiScan	3Seq
NParks/M54-9 (accession number MW194928)	5239–5357	GI.4; accession number KX357697^a^	GI.2; accession number KF442962^b^	2.807 × 10^−83^	1.087 × 10^−79^	9.188 × 10^−86^	3.479 × 10^−30^	1.863 × 10^−31^	1.456 × 10^−39^	1.133 × 10^−10^

a 92.7 per cent identity to the Australian RHDV strain (KX357697). ^b^96.8 per cent identity to the Portugal RHDV strain (KF442962). CI = confidence interval.

**Figure 2.**
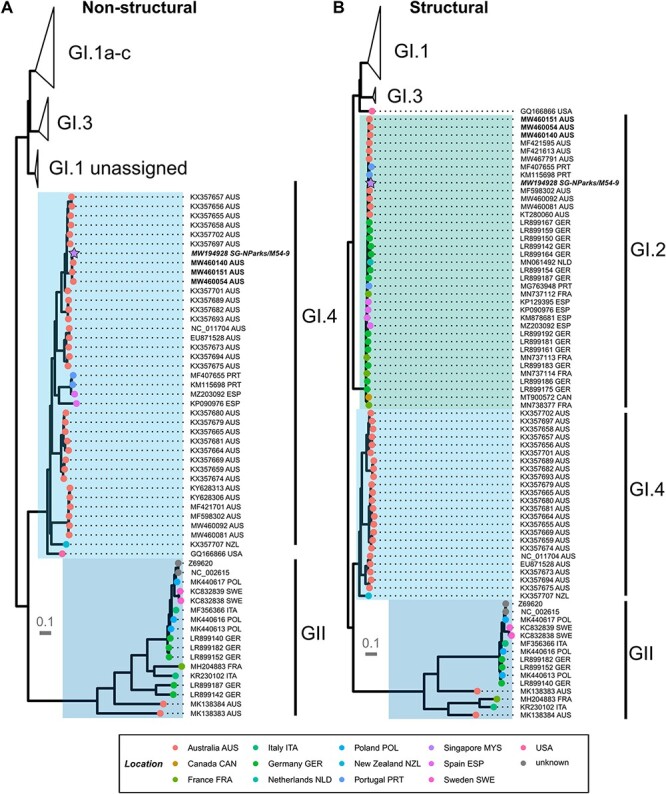
Maximum-likelihood phylogenies of representative global lagovirus sequences. Phylogenies were estimated in IQTREE2 using the best-fit model as selected by ModelFinder and were estimated separately for the (A) NS and (B) S sequences. The GI.4cP-GI.2 viruses are identified in bold, while the Singapore RHDV 2020 sequence (NCBI accession number MW194928) is presented in bold italics. Branch support was estimated with 1,000 ultrafast bootstrap replicates and 1,000 replicates of the SH-aLRT test. The scale bar shows nucleotide substitutions per site. Tips were coloured based on the location from which the sequence was reported. Highlighted regions show (from bottom to top) genogroup GII , genotype GI.4 , genotype GI.2 , genotype GI.1 , and genotype GI.3 . One sequence is unclassified in the structural regions and has not been highlighted. GI.1 and GI.3 clades have been collapsed.

When checked against the NCBI nr/nt database using blastn, the sequence used in this study showed 99.2 per cent sequence identity across the full genome to the Australian RHDV2 GI.4cP-GI.2 (nomenclature definition as [RdRp genotype]P − [capside genotype]) KEI-2 virus (GenBank accession MW460111) as reported by Mahar et al. (2021). Australian GI.4cP-GI.2 sequences could be subdivided into five distinct lineages (i to v) (Mahar et al. 2021); the nine closest blastn matches to the Singapore RHDV 2020 sequence were all from lineage i of the GI.4cP-GI.2 viruses. Our phylogenetic analyses showed that the 2020 Singapore RHDV sequence was closely related to Australian GI.2 and two Portuguese viruses in the S phylogeny and clearly clustered within the diversity of Australian GI.4cP sequences in the NS phylogeny (Fig. 2). This further supports an Australian origin for the 2020 Singapore RHDV strain incursion in the same year.

### Time-structured phylogeographic analysis

**Figure 3.**
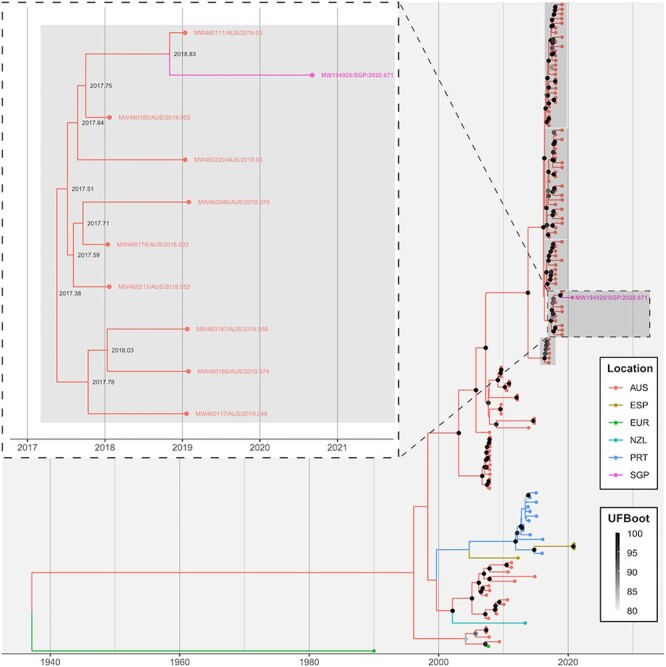
The phylogeography of RHDV2 VP60 sequences. A time-structured maximum-likelihood phylogeny was inferred for all non-Australian full-length lagovirus GI.2 VP60 sequences available through GenBank where a collection date and location were available, along with the selected Australian GI.2 VP60 sequences (1,743 nt; *n* = 293). EBHSV (NC_002615/EUR/1990.003) was used as an outgroup. Tips are coloured based on sampling location. The ancestral state reconstruction was performed to infer the location of branches. Branch support was estimated using 1,000 iterations of the SH-aLRT test and 1,000 ultrafast bootstrap (UFBoot) approximations. For visualisation purposes, only UFBoot values >80 are shown as grey points at internal nodes. The inset shows the immediate clade within which the sequence of interest, MW194928/SGP/2020.671, falls. The GI.4cP-GI.2 clades have been highlighted in grey in both the main tree and the insets. The taxa name for this sequence is given in both the main figure and the inset. The inferred dates of internal nodes are shown in the inset. AUS, Australia; CHN, China; ESP, Spain; EUR, Europe; FRA, France; MAR, Morocco; NLD, the Netherlands; POL, Poland; PRT, Portugal; SGP, Singapore; TUN, Tunisia.

**Figure 4.**
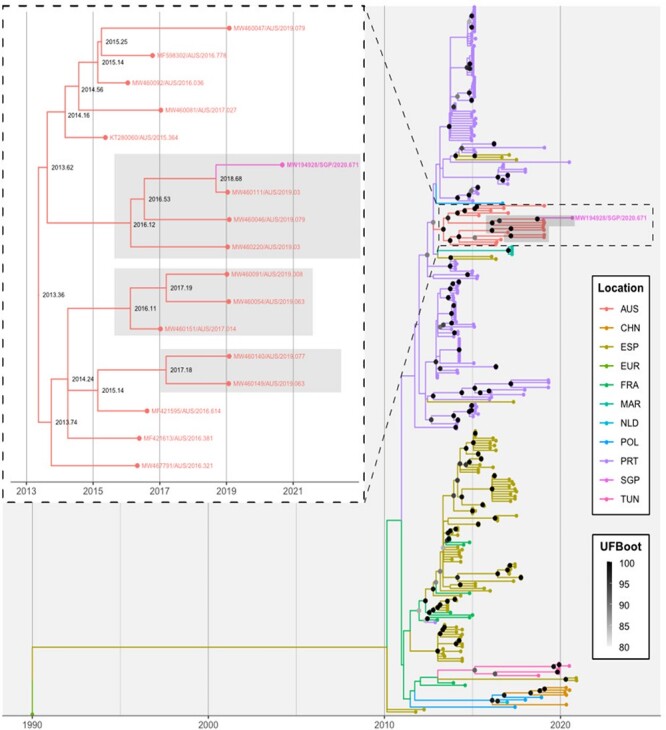
The phylogeography of RHDV2 non-structural sequences. A time-structured maximum-likelihood phylogeny was inferred for all full-length lagovirus GI.4 NS sequences available through GenBank, where a collection date and location were available (5,268 nt; *n* = 140). EBHSV(NC_002615/EUR/1990.003) was used as an outgroup. Tips are coloured based on sampling location. The ancestral state reconstruction was performed to infer the location of branches. Branch support was estimated using 1,000 iterations of the SH-aLRT test and 1,000 ultrafast bootstrap (UFBoot) approximations. For visualisation purposes, only UFBoot values >80 are shown; these are shown as grey points at internal nodes. The inset shows the immediate clade within which the sequence of interest, MW194928/SGP/2020.671, falls. The GI.4cP-GI.2 clades have been highlighted in grey in both the main tree and the insets. The taxa name for this sequence is given in both the main figure and the inset. The inferred dates of internal nodes are shown in the inset. AUS, Australia; ESP, Spain; EUR, Europe, NZL, New Zealand; PRT, Portugal; SGP, Singapore.

Root-to-tip regression identified a strong clock-like signal in the GI.2 VP60 S (*r*^2^ = 0.82) data set (Fig. 3) although this was weaker in the GI.4 NS (*r*^2^ = 0.39) data set (Fig. 4). The evolutionary rate was estimated at 3.2 × 10^–3^ (±1.4 × 10^–4^) and 4.5 × 10^–3^ (±1.2 × 10^–4^) substitutions per site per year. As in the distance-based phylogeny, the Singapore RHDV2 sequence distinctly clustered with Australian sequences in both time-structured analyses with strong branch support (Figs. 3 and 4). The most closely related sequence in both the NS and VP60 S time-structured analyses was MW460111, noting that this sequence was not used in the distance-based phylogeny because of the methodology employed to select samples for inclusion for that analysis. The estimated divergence date from the most closely related sequence was early September 2018 (2018.68, 90 per cent maximum posterior region 2018.22–2018.94) for the GI.2 VP60 S data set (Fig. 3) and late October 2018 (2018.83, 90 per cent maximum posterior region 2018.67–2018.91) for the GI.4 NS data set (Fig. 4).

## Discussion

Recombination events contribute to genetic diversity in many viruses; in caliciviruses, the major recombination breakpoint corresponds to the cleavage site of the capsid gene during post-translational processing (Bull et al. 2005; Forrester et al. 2008). Recombination of GI.2 S sequences with both non-pathogenic and pathogenic variant NS regions has been reported, demonstrating not only the importance of recombination in increasing GI.2 diversity but likewise the high recombination capability within lagoviruses (Lopes et al. 2015; Mahar et al. 2021). The GI.2 variant was observed to be able to infect non-*Oryctolagus* lagomorph hosts (Velarde et al. 2017), cause lethal disease in young kits, and even overcome RHDV immunity (Dalton et al. 2012; Le Gall-Reculé et al. 2013). Indeed, it is the GI.2 S regions that are the primary determinants of host range, antigenicity, and pathogenicity (Mahar et al. 2021). The recombinant GI.2 (RHDV2/RHDVb) variant reported in this study revealed a pathogenic GI.2 and a non-pathogenic GI.4 variant, with nucleotide homology most closely related to similar RHDV2/GI.4c variants identified in Australian rabbit and hare populations (Mahar et al. 2021).

Australian GI.4cP-GI.2 variants were first detected in early 2017; however, the phylodynamic analysis revealed the emergence of at least five independent GI.4cP-GI.2 recombinant lineages (i to v) between 2016 and 2017 (Mahar et al. 2021). Phylogeographic analyses in this study clearly identified Australia as the most likely ancestor emergence location for the Singapore RHDV2 sequence. This was despite deliberately restricting the number of Australian sequences in the GI.2 VP60 analysis to mitigate oversampling bias. This is not surprising, given that the recombinant GI.4cP-GI.2 variant was reported to have emerged in Australia relatively recently, from late 2015 to early 2017 (Mahar et al. 2021). This variant has not been reported in any other country. Furthermore, we could show that the 2020 Singapore RHDV sequence falls within sublineage i of the GI.4cP-GI.2 RHDVs that first emerged in Victoria, most likely in early 2017, and subsequently spread to New South Wales and South Australia. This lineage was not present in Queensland, Tasmania, Western Australia, or the Northern Territory in 2020. This strongly suggests that the Singapore incursion occurred via the southern or south-eastern mainland states of Australia.

Strikingly, the estimated time to the most recent common ancestor (TMRCA) of the Singapore RHDV2 and Australian RHDV2 sequences was very similar across the GI.4 NS and GI.2 VP60 datasets, approximately from early September to late October 2018 (combined 90 per cent maximum posterior region ranging from 2018.22 to 2018.94). The fact that these estimates were derived based on a single sequence from the Singapore outbreak and yet still align closely strongly supports this assumption. Furthermore, the similarity in TMRCA across the two data sets suggests a shared evolutionary history between the two genetic regions, i.e. no further recombination events in unsampled ancestors of the Singapore sequence.

The evolutionary rates estimated in our analysis were broadly similar across both GI.4 NS and GI.2 VP60 data sets and are comparable to those previously reported for lagoviruses, which have ranged from 2.8 × 10^–3^ to 5.7 × 10^–3^ substitutions per site per year (Eden et al. 2015; Mahar et al. 2016, 2018).

A separate epidemiological study into the spread of the virus within Singapore reported local transmission; the introduction of RHDV into Singapore by the importation of infected rabbits and rabbit feed was, however, ruled out, due to restricted international movements at the time of the outbreak (due to the COVID19 pandemic), a lack of detection of new cases after heightened passive biosurveillance, and strict legislations imposed by the country (Lim et al. 2021). Henning et al. (2005) reported that RHDV is extremely environmentally stable, remaining viable in animal tissues (e.g. rabbit carcasses) for more than 90 days and on other fomites (e.g. materials contaminated with excretions) for a month (Henning et al. 2005), which could suggest potential transmission through contaminated objects from Australia. Phylogenetic analyses clearly show that the source of this virus was the Australian lagomorph population although the mechanism by which the RHDV2/GI.4 strain was introduced into Singapore still requires further elucidation.

Biosecurity and immunoprophylactic measures are still the ‘gold standard’ for limiting the spread of RHDV in rabbits (Abrantes et al. 2012). As of 30 November 2020, no new RHDV cases had been reported in Singapore (Toh et al. 2021). For the rapid and sensitive detection of acute RHD, detection by reverse-transcription quantitative PCR is most useful (Toh et al. 2021). Serological tests based on the VP60 gene are available for RHDV (Marchandeau et al. 2005; Lavazza and Capucci 2008); however, antibodies are only present in surviving animals and are not immediately detectable during acute infection (Hall et al. 2021). With the increasing understanding of calicivirus recombination, new enzyme-linked immunosorbent assay (ELISA) tests and real-time PCR testing methods will need to be developed to identify the variant(s) more specifically. Currently, there is no specific treatment available for rabbits infected with RHDV although commercial vaccines are available for domesticated rabbits against RHDV1 and RHDV2. The development of improved diagnostics (for instance, differential diagnostic real-time PCR based on the S protein) and vaccines will also need to be continuously evaluated to confer the timely disease detection and identification of novel viral recombinants and protection to lagomorphs.

## Supplementary Material

vead029_Supp
